# Inadequate methods undermine a study of malaria, deforestation and trade

**DOI:** 10.1038/s41467-021-22514-4

**Published:** 2021-06-18

**Authors:** Nikolas Kuschnig

**Affiliations:** Vienna University of Economics and Business (WU), Vienna, Austria

**Keywords:** Ecological epidemiology, Environmental economics, Statistics

**Arising from** Chaves et al. *Nature Communications* 10.1038/s41467-020-14954-1 (2020)

In a recent study, Chaves et al.^1^ find international consumption and trade to be major drivers of ‘malaria risk’ via deforestation. Their analysis is based on a counterfactual ‘malaria risk’ footprint, defined as the number of malaria cases in absence of two malaria interventions, which is constructed using linear regression. In this letter, I argue that their study hinges on an obscured weighting scheme and suffers from methodological flaws, such as disregard for sources of bias. When addressed properly, these issues nullify results, overturning the significance and reversing the direction of the claimed relationship. Nonetheless, I see great potential in the mixed methods approach and conclude with recommendations for future studies.

To construct ‘malaria risk’, Chaves et al.^1^ regress malaria cases on cumulative tree cover loss and two malaria intervention variables, expressed in shares of usage. Their globally aggregated data cover the period from 2000 until 2015 on a yearly basis. Data on malaria cases and tree cover loss are available for 26 countries in tropical biomes, while the two intervention variables are only available for 13 of these countries in Africa. Figure 1 shows the time series under scrutiny; additional information on the data is provided in Supplementary Note 1.

**Fig. 1:**
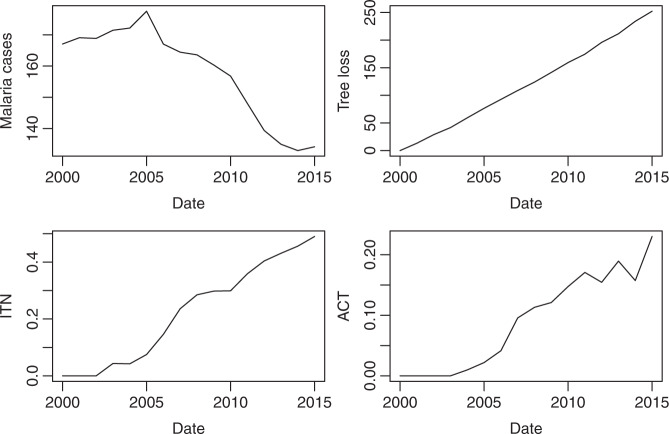
Time series under consideration. Variables are malaria cases (in million), cumulative tree cover loss (in million hectare), percent sleeping under insecticide-treated nets (ITN) and percent of under-5 fevers receiving artemisinin-based combination therapies (ACT).

Chaves et al.^1^ specify their regression model as (see their paper for notation)

1 $$\mathop{\sum }\limits_{r}{I}_{r}(t)={\beta }_{0}+{\beta }_{L}\mathop{\sum }\limits_{r}{L}_{r}(t)+{\beta }_{n}n(t)+{\beta }_{a}a(t).$$

However, the actual model is a weighted regression of the type

2 $$w(t)\mathop{\sum }\limits_{r}{I}_{r}(t)={\beta }_{0}+{\beta }_{L}w(t)\mathop{\sum }\limits_{r}{L}_{r}(t)+{\beta }_{n}w(t)n(t)+{\beta }_{a}w(t)a(t)+\epsilon (t),$$

where *w*(*t*) is a weight scalar and *ϵ*(*t*) is an error term at time *t*. Weights were constructed via replication of observations, meaning that ∑_*t*_*w*(*t*) ≠ 1. The sample size is not adjusted accordingly, meaning that standard errors are too small by a factor of 2.08 on average (see Table 1, column two). The weighting was obscured by its omission from the Methods and by the replicated rows only being visible after unhiding them in the spreadsheet that is provided in their replication files. Chaves et al.^1^ weigh 2005 at 42.86%, 2001 at 17.86%, and 2014 at 16.07%. The unweighted model, as it is specified in the paper, undoes the significance and switches the sign of forest loss, as can be seen in columns one and three of Table 1.

**Table 1:** Comparison of original regression results to alternatives.

Malaria cases	(1)	(2)	(3)	(4)	(5)
Constant	170.170***	169.414***	176.315***	173.726***	0.092
	(1.780)	(3.914)	(4.025)	(1.379)	(0.341)
Tree loss	0.306***	0.321**	−0.057	−0.463***	−0.047
	(0.051)	(0.113)	(0.132)	(0.116)	(0.054)
ITN	−279.220***	−285.038***	−52.356	−186.717***	−68.360***
	(37.959)	(82.012)	(81.002)	(30.347)	(21.913)
ACT	135.634**	136.685	2.249	76.393*	32.654
	(60.590)	(129.038)	(117.189)	(44.443)	(23.487)
Time				10.113***	
				(1.441)	
*N*	56	16	16	56	55
*R*^2^	0.915	0.911	0.827	0.957	0.326

Column (1) holds the reproduced regression. Column (2) corrects duplicated observations and sample size, leading to increased standard errors. Column (3) removes the weighting scheme. Column (4) includes time as explanatory variable, demonstrating issues with omitted variables and stationarity. Column (5) models the dynamic relation of variables by considering yearly changes of all variables. Note that only single adaptations are made and other issues remain present. Standard errors in (brackets).

*p ≤ 0.1; **p ≤ 0.05; ***p ≤ 0.01.

The study by Chaves et al.^1^ is looking to estimate a causal effect of deforestation on malaria incidence. Valid estimates of this relation can only be obtained using appropriate techniques and assumptions that require theoretical justification^2^. The authors do not consider these intricacies and offer no explanation of why their ‘malaria risk’ measure may be interpreted as it is. Instead, they disregard a number of statistical issues that I discuss below.

Chaves et al.^1^ base their model selection on achieving a ‘sufficient’ *R*^2^—a procedure that is well known to be inadequate^3^. To illustrate this, consider a regression of birth rates on stork population. Common seasonal patterns lead to high correlation and high values of *R*^2^. However, we learn very little about the actual relationship and estimates will be spurious. Chaves et al.^1^ claim that any model adaptation would only marginally increase *R*^2^ and hence necessarily mimic their results. This is factually incorrect, missing the relative nature of *R*^2^. See column (4) of Table 1 for a demonstration of how an additional variable can affect results.

Obtaining unbiased estimates from a linear regression relies on the exogeneity assumption, i.e. no correlation between explanatory variables and the error term. This assumption is commonly violated by simultaneity or omitted variables^4^. Simultaneity occurs when variables are determined contemporaneously, e.g. due to reciprocal causation. Regressing a disease’s incidence on its interventions is a textbook example for this phenomenon. Valid inference could only be drawn using elaborate methods, such as instrumental variables, or, if theoretically justifiable, by assuming no effects of malaria incidence on the use of nets and therapy. Omitted variable bias occurs when the dependent and explanatory variables are both affected by a third factor. Chaves et al.^1^ cite Garg^5^ and Berazneva and Byker^6^, who establish causal links between deforestation and malaria for specific regions. These studies rely on panel data, allowing for subnational heterogeneity, and an extensive set of control variables in order to distil a causal effect. Chaves et al.^1^ themselves observe a number of malaria determinants in their appendix, which are also drivers of deforestation^6^. Yet, the authors do not take any of these factors into account. The distortion caused by this oversight becomes noticeable when including a linear time trend, as one of many omitted variables (see Table 1, column (4)).

In their study, Chaves et al.^1^ perform a time series regression without considering any of the associated complexities. Crucially, their model relies on stationarity of variables, i.e. their distributions, hence moments such as the mean, must be constant over time^4^. Non-stationary variables generally lead to the spurious regression problem^7^. Results would then indicate strong correlation between variables, but do not imply causation. In the study’s model, we cannot reject non-stationarity for any of the variables considered and we find autocorrelated residuals—all at any reasonable level of significance (see Supplementary Table 1 for test results). The variable of interest, cumulative forest loss, is even non-stationary by design. When dealing with this issue in two simple ways, we find completely different results—namely sign-switching and insignificant coefficients. See columns (4) and (5) of Table 1 for a model accounting for a linear time trend and one where the relation of yearly changes of variables is modelled.

Putting aside inadequate methods, there is a number of simplifications that neglect important complexities of both malaria and deforestation dynamics. By aggregating data, Chaves et al.^1^ implicitly assume international homogeneity of malaria dynamics. This assumption is striking, given weak empirical support^8^ and the spatial mismatch of malaria and forest loss. Malaria predominantly occurs in Africa, with 93% of global cases in 2018^9^, while forest loss mostly stems from other regions^10^. Furthermore, Chaves et al.^1^ silently equate the distinct concepts of forest loss, deforestation and commodity-driven deforestation. With the Hansen et al.^10^ data, they use information on forest loss, which is only partly due to deforestation^10,11^. Deforestation, in turn, is driven by multiple factors, including but not limited to commodity production^12^. Since commodity-driven deforestation is only a subset of forest loss, with arguably special dynamics, this distinction is relevant for conclusions that can be drawn.

To sum up, the study by Chaves et al.^1^ constitutes an important attempt at linking malaria, deforestation and trade, but falls short of this ambitious goal. Their use of an unorthodox weighting scheme lacks justification and pushes results towards showing a link between deforestation and malaria. Their model is plagued by a number of serious methodological issues, including simultaneity, omitted variables and non-stationarity. Each one of them individually is enough to invalidate results. Still, I hope this direction is pursued further and offer some recommendations: (a) be transparent with assumptions made, (b) approach interdisciplinary problems with an interdisciplinary team, (c) be precise and careful with the notion of causality.

## Supplementary information

Supplementary Information

Description of Additional Supplementary Files

Supplementary Software 1

## Data Availability

All data used for this work stem from the original research paper by Chaves et al.^1^ and can be found in their online repository at 10.5281/zenodo.3630653.

## Code availability

All code used for this work can be found in Supplementary Software 1 or online at https://gist.github.com/nk027/44af20da3e337f69e0052870ef21e8ed.
