# Supplementary material for: Inadequate methods undermine a study of malaria, deforestation and trade
Source: Nat Commun. 2021 Jun 18;12:3762. doi: 10.1038/s41467-021-22514-4 (PMC8213779; doi:10.1038/s41467-021-22514-4)
Supplement: Supplementary file 1 — Supplementary Information [file 41467_2021_22514_MOESM1_ESM.pdf]

Supplementary Information

Inadequate methods undermine a study of malaria, deforestation and trade

Nikolas Kuschnig

Supplementary Note 1

Data is aggregated by summing up malaria cases and tree cover loss, and averaging the intervention shares. This implies equal weights for all countries, meaning that the intervention shares for countries like Nigeria (61 million malaria cases in 2015) and Gabon (401 thousand malaria cases in 2015) have equal influence.

Both malaria intervention variables only cover 13 of the 26 total countries, all of which are located in Africa. This means that there is no data on malaria interventions for Brazil and Indonesia, the countries with the largest amount of forest loss.

In the spreadsheet that is provided in the replication files some of the duplicated observations for 2001 are unique. The reason for this is not clear, but values appear to stem from combinations of explanatory variables from between 2000 and 2004.

The inflation of standard errors and hence t values is due to the increased sample size and can be calculated by comparing the factors $\sqrt{N - DoF}$ for the erroneous sample size 56 and the corrected one at 16.

Supplementary Table 1

Table 1: Test statistics for stationarity. The augmented Dickey-Fuller tests fail to reject a unit-root (or non-stationarity). The Phillips-Ouliaris test fails to reject no-cointegration of variables. The Durbin-Watson test rejects no-autocorrelation of residuals.

	statistic	p value
Augmented Dickey-Fuller test (malaria cases)	-2.439	0.405
Augmented Dickey-Fuller test (tree cover loss)	0.259	> 0.99
Augmented Dickey-Fuller test (ITN)	-2.124	0.525
Augmented Dickey-Fuller test (ACT)	-2.508	0.379
Phillips-Ouliaris test	-4.023	0.15
Durbin-Watson test	0.493	< 0.01

Supplementary Methods 1

Please find an R script to reproduce the letter's results in the Supplementary Software 1 or online at https://gist.github.com/nk027/44af20da3e337f69e0052870ef21e8ed. Note that the script relies on the original replication file at https://doi.org/10.5281/zenodo.3630652.
