# Supplementary material for: Inadequate methods undermine a study of malaria, deforestation and trade
Source: Nat Commun. 2021 Jun 18;12:3762. doi: 10.1038/s41467-021-22514-4 (PMC8213779; doi:10.1038/s41467-021-22514-4)
Supplement: Supplementary file 2 — Description of Additional Supplementary Files [file 41467_2021_22514_MOESM2_ESM.pdf]

Description of Additional Supplementary Files

File Name: Supplementary Software 1
Description: R Script to reproduce results from the letter.
